# Functional Genomic Analysis of *C. elegans* Molting

**DOI:** 10.1371/journal.pbio.0030312

**Published:** 2005-08-30

**Authors:** Alison R Frand, Sascha Russel, Gary Ruvkun

**Affiliations:** **1**Department of Molecular Biology, Massachusetts General Hospital, Boston, Massachusetts, United States of America, and Genetics Department, Harvard Medical School, Boston, Massachusetts, United States of America; Utrecht UniversityNetherlands

## Abstract

Although the molting cycle is a hallmark of insects and nematodes, neither the endocrine control of molting via size, stage, and nutritional inputs nor the enzymatic mechanism for synthesis and release of the exoskeleton is well understood. Here, we identify endocrine and enzymatic regulators of molting in *C. elegans* through a genome-wide RNA-interference screen. Products of the 159 genes discovered include annotated transcription factors, secreted peptides, transmembrane proteins, and extracellular matrix enzymes essential for molting. Fusions between several genes and green fluorescent protein show a pulse of expression before each molt in epithelial cells that synthesize the exoskeleton, indicating that the corresponding proteins are made in the correct time and place to regulate molting. We show further that inactivation of particular genes abrogates expression of the green fluorescent protein reporter genes, revealing regulatory networks that might couple the expression of genes essential for molting to endocrine cues. Many molting genes are conserved in parasitic nematodes responsible for human disease, and thus represent attractive targets for pesticide and pharmaceutical development.

## Abstract 2

The authors use a genome-wide RNA-interference screen to identify and characterize genes involved in C. elegans molting. They investigate regulatory networks involved in molting, lending important new insights into this complex process.

## Introduction

Ecdysozoan animals, including nematodes and arthropods [1], develop through periodic larval stage molts when the exoskeleton is shed and synthesized anew. Although molting is the hallmark of the most abundant and diverse group of animals on the planet, including a wide variety of human pests and pathogens, the endocrine circuits that regulate molting in response to environmental and physiologic cues are not well understood. Moreover, little is known about the molecular mechanisms for release and de novo production of the exoskeleton.

Endocrine and neuroendocrine pathways regulate molting in arthropods, and likely operate in nematodes as well. In insects, pulses of the steroid hormone ecdysone trigger molting and metamorphosis [2,3]. The neuropeptide prothoracicotropic hormone stimulates synthesis of ecdysone in the prothoracic glands [3]. At the end of each larval stage, the neuropeptide eclosion hormone, combined with a decline in the titer of ecdysone, prompts release of the peptide ecdysis-triggering hormone from glands lining the trachea [4–7]. Ecdysis-triggering hormone then promotes behaviors essential for escaping the old exoskeleton [8,9], and also stimulates neurons to secrete more eclosion hormone, creating a positive feedback loop that culminates in a hormonal surge decisive for ecdysis [10]. Environmental cues, including photoperiod, temperature, and humidity, as well as physiologic factors, including size, stage, and the nutritional status of the organism, modulate secretion of prothoracicotropic hormone in various arthropods, suggesting extensive sensory input to the neuroendocrine secretions that govern molting [3]. However, little is known about the circuits that initiate, terminate, or set the pace of the molting cycle in any Ecdysozoan.

Although an endocrine trigger for nematode molting has yet to be identified, several lines of evidence implicate steroid hormones in *Caenorhabditis elegans* molting. Molting of *C. elegans* requires cholesterol, the biosynthetic precursor of all steroid hormones, as well as the low-density lipoprotein (LDL) receptor-like protein LRP-1, which is thought to endocytose sterols from the growth medium [11]. A sterol-modifying enzyme synthesized in the intestine, LET-767, is also essential for molting, consistent with the production or modification of a hormone derived from steroids [12]. The best evidence of a hormonal cue for molting of *C. elegans* is the requirement for two nuclear hormone receptors (NHRs), NHR-23 and NHR-25, orthologous, respectively, to the ecdysone-responsive gene products DHR3 and Ftz-F1 of *Drosophila melanogaster* [13–16]. Ecdysone itself, however, is unlikely to serve as a molting hormone in nematodes because ecdysteroids have not been detected in any free-living nematode [17], and because orthologs of the ecdysone receptor components EcR and USP (Ultraspiracle) have not been identified in the complete genome of *C. elegans* [18].

Molting of *C. elegans* involves the synthesis and secretion of a new exoskeleton underneath the old one, separation of the old exoskeleton from the epidermis (apolysis), and escape from the old exoskeleton (ecdysis) [19]. At the end of each stage, larvae become inactive for a brief period of time known as lethargus that coincides with separation of the old exoskeleton from the epidermis. Next, particular behaviors promote ecdysis; larvae flip on their long axis to loosen the body cuticle, expel the anterior half of the pharyngeal cuticle, and ultimately escape the old exoskeleton via a forward thrust [19].

The exoskeleton of nematodes, called the cuticle, is a collagenous extracellular matrix secreted by underlying epithelial cells, known as the hypodermis and seam cells, and also by specialized interfacial cells that line openings of the body, including the buccal cavity, pharynx, vulva, rectum, and sensilia [20]. Lipids and glycolipids comprise the outermost layer of the cuticle, whereas glycoproteins, thought to be secreted by gland cells, form the surface coat [21]. Elasticity of the cuticle permits growth during each larval stage, but particular structures, such as the buccal cavity, grow saltationally at molts [22]. The distinction between collagen in the nematode exoskeleton and chitin in the insect exoskeleton suggests that the enzymatic cascades that mediate release of the exoskeleton in nematodes may be distinct from those that release the exoskeleton in arthropods. Although two collagenases essential for molting have been identified in *C. elegans* [23,24], the full ensemble of signaling proteins and extracellular matrix enzymes required to remodel the exoskeleton has yet to be illuminated.

Human diseases caused by parasitic nematodes affect tropical regions of Africa, Asia, and South America. The World Health Organization estimates that 120 million people endure lymphatic filariasis (elephantiasis), due to infection by the filarial nematodes *Wuchereria bancrofti* or *Brugia malayi,* and that 18 million people endure onchocerciasis (African river blindness), due to infection by *Onchocerca volvulus* [25]. *Ascaris,* hookworms, and whipworms are also important pathogens, infecting approximately 1 billion people. Parasitic nematodes further damage livestock and lay waste to $80 billion of crop plants annually. One promising approach to the discovery of new targets for anti-nematode drugs, vaccines, and pesticides is the identification of nematode-specific genes essential for the viability of larvae. In the screen described here, a large number of nematode-specific genes essential for molting were identified, and some encode attractive drug targets.

Here, we identify endocrine and enzymatic regulators of molting in *C. elegans* through a genome-wide RNA-interference (RNAi) screen, providing a broad view of functions essential for molting in a model Ecdysozoan. We further develop models for the genetic regulation of molting based on the location, timing, and order of expression of particular molting genes.

## Results

**Figure 1:**
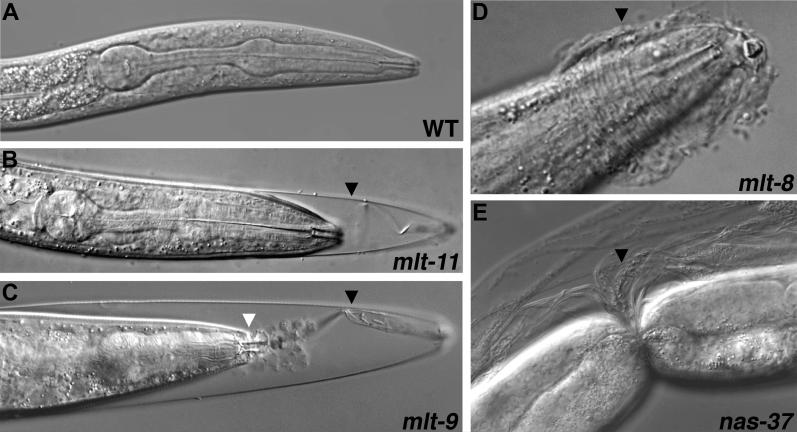
Molting Defects Caused by RNAi N2 larvae were fed bacteria expressing dsRNA corresponding to the indicated genes, or control bacteria not expressing dsRNA of a worm gene (A). Panels A–D show the anterior, whereas (E) shows the mid-body of a larva. Black arrowheads mark unshed cuticle. White arrowhead (C) indicates the buccal capsule. Nomarski optics.

To identify a full set of endocrine and enzymatic regulators of molting in *C. elegans,* we screened a combined library of 18,578 bacterial clones that each express a double-stranded RNA designed to silence one of the 19,427 predicted worm genes via RNAi [26–28]. About 25 L1-stage larvae were fed each clone and later examined for molting defects, indicated by the adherence of cuticle from the pre-molt larval stage to the body of the worm (the Mlt phenotype; Figure 1). Gene inactivations observed to prevent molting in the primary library screen were tested again by feeding the bacterial clones to approximately 50 wild-type (N2) and 50 *rrf-3(pk1426)* mutant larvae, a genetic background where RNAi is more effective [29].

Inactivation of 159 genes (Tables 1 and S1–S4) interfered with molting. Tables 1 and S1 show genes whose inactivation produced molting defects in 10% to 100% of wild-type (N2) or *rrf-3(pk1426)* mutant larvae. Eighty-seven other genes were assigned a lower priority based on gene annotation (Tables S2 and S3) or the low penetrance of molting defects observed after RNAi (Table S4). The blind identification of nine genes previously described to cause an arrest at a molt, including *lrp-1, nhr-23, nhr-25, nas-37, nas-36, skp-1, rme-8, acn-1,* and *bli-4* [11,13–16,23,24,30–33], verified the efficacy of this RNAi-based strategy for isolating bona fide molting genes. An additional 28 of the 159 gene inactivations were independently described as causing an arrest at a molt in broad screens that identified many loss-of-function phenotypes using RNAi [26,27,34]. Further, the observation of molting defects in larvae with mutations in *qua-1* (unpublished data), *lrp-1* [11], and *nas-37* [23] verified that RNAi faithfully recapitulates loss-of-function phenotypes in the molting pathway. The names *mlt-8, mlt-9, and mlt-11* were assigned, respectively, to *W08F4.6, F09B12.1*, and *W01F3.3* after expression data verified a primary function in molting. The genes *fbn-1, noah-1,* and *noah-2* were assigned names based on homology to genes of mammals or insects (Table S1).

**Table 1:**
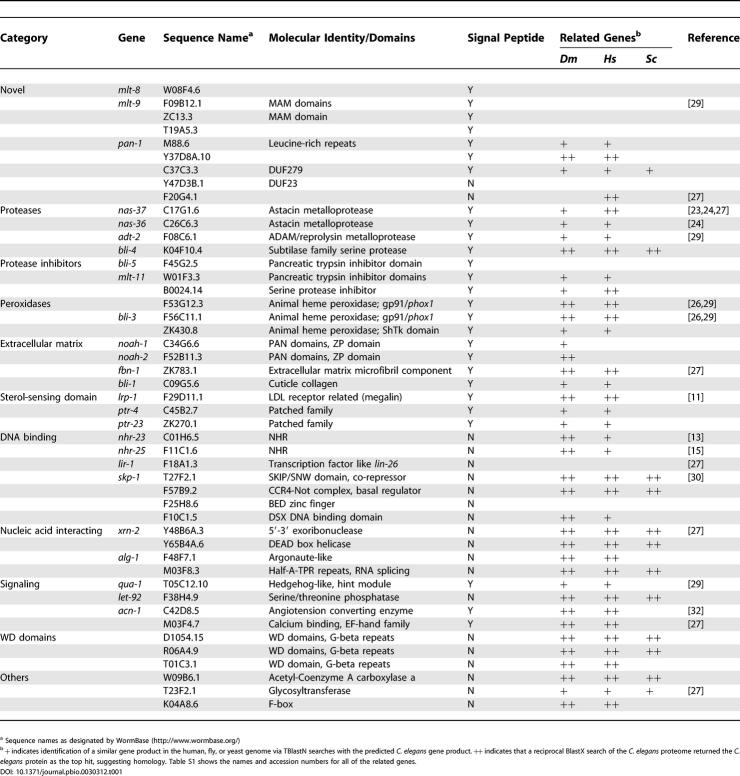
Selected Genes Whose Inactivation Disrupts Molting

Figure 1 shows examples of molting-defective larvae produced by RNAi. Most often, larvae were observed incarcerated in sheaths of old cuticle extending from the anterior end of the worm, as shown for a *mlt-11(RNAi)* larva (Figure 1B). The nature of molting defects caused by particular gene inactivations suggested that the corresponding proteins function in a specific anatomical place or stage of ecdysis. For example, L4-stage larvae fail to shed cuticle from the pharynx after RNAi of *xrn-2* (unpublished data). A similar type of molting defect has been observed in animals lacking the DNA binding protein PEB-1 [35]. Many *mlt-9(RNAi)* larvae fail to shed cuticle lining the buccal cavity, causing the lips to evert (Figure 1C). Unshed cuticle often forms coronal constrictions on *nas-37(RNAi)* larvae (Figure 1E), possibly when animals flip on their long axis during molting. Inactivation of the collagenase gene *nas-37* also prevents the breakdown of old cuticle at the anterior tip of the worm, thereby blocking escape from the old exoskeleton (unpublished data) [23,24]. Although Mlt larvae typically arrest development, some gradually escape from the old cuticle, only to fail again at the next molt, a phenomenon observed often after RNAi of *qua-1* (unpublished data).

The majority of genes we identified are likely to act at all four molts, because their inactivation prevents molting from several larval stages. Moreover, although feeding L1-stage larvae dsRNA for particular genes, such as *mlt-8* or *acn-1,* prevents development beyond the L3 stage (Table S5), feeding the same dsRNAs to older larvae also disrupts the final molt (unpublished data). The majority of gene inactivations also disrupt molting from the dauer stage, an alternative L3 stage that is adapted for survival in unfavorable conditions and resembles the infective form of parasitic nematodes (Table S6).

The majority of genes we identified are conserved in parasitic nematodes responsible for human, animal, and plant diseases (Table S7). Many of the genes, including *mlt-8* and *mlt-9,* are conserved only in nematodes; similar proteins are readily identified among the predicted products of cDNAs or genomic sequences from parasitic and free-living nematodes (Table S7), but not in the translated genomes of *D. melanogaster* or *Homo sapiens* (Table S1). In contrast, the genes *noah-1* and *noah-2,* which specify putative extracellular matrix components, are conserved in insects and nematodes, but not in humans, and thus show the phylogenetic conservation signature expected for molting genes common to Ecdysozoans.

### Predicted Functions of Genes Uncovered in the Molting Screen

In this section, we discuss how the annotations of particular genes uncovered by RNAi implicate the corresponding proteins in basic aspects of the molting cycle. Based on experimental evidence of a steroidal pathway, as well as the evolutionary relationship between arthropods and nematodes [1], we expect that endocrine cues periodically initiate molting in *C. elegans,* stimulating the synthesis of a new cuticle and release of the old one. We expected to isolate many genes essential for apolysis or ecdysis because we screened for larvae arrested at the final stage of the molt. However, we also anticipated the identification of genes required for the production of, or response to, hormonal cues for molting, because the loss of either *nhr-23* in *C. elegans* or *EcR* in *D. melanogaster* can result in a terminal failure to ecdyse [14,36]. Also, a breakdown in the coordination of signaling events associated with molting might trigger an aberrant ecdysis and thereby cause arrest at that stage.

### Regulation of Gene Expression

The identification of several transcription factors suggests that molting of *C. elegans* requires extensive changes in gene expression, similar to how transcriptional cascades promote molting and metamorphosis of insects [2]. Particular transcription factors likely alter gene expression in epithelial cells, possibly in response to endocrine cues. Annotated DNA binding proteins and transcription factors required for molting include three zinc-finger proteins, specified by *F10C1.5, F25H8.6,* and *lir-1* [37], that resemble, respectively, *Drosophila* Doublesex, BED subfamily members, and the *C. elegans* transcription factor LIN-26, as well as two NHRs, NHR-23 and NHR-25, that were previously implicated in molting [13,15]. NHR-23 and NHR-25 are the best candidates for transducing hormonal signals, because the NHRs are expressed in epithelial cells and conserved in insects [13,15,16,30]. In theory, NHRs required for molting might regulate the expression of zinc-finger transcription factors identified in this screen, just as particular NHRs activate zinc-finger proteins in the transcriptional cascades coupled to insect metamorphosis [2].

The *xrn-2* gene encodes a 5′-3′exoribonuclease that is conserved from yeast to humans [38] and is essential for molting in *C. elegans*. The homologous enzyme, Rat1p, is required for degradation of nuclear pre-mRNAs as well as the 5′ processing of ribosomal and small nuclear RNAs in *Saccharomyces cerevisiae* [39–41]. Consistent with a role for *C. elegans* XRN-2 in gene regulation, the degradation pathway mediated by Rat1p in yeast is known to compete with productive mRNA splicing [41].

### Intercellular Signaling

Probable signaling components were identified in the molting screen, consistent with expectations of an endocrine cue for molting, coordination of the process in different cell types, and physiologic feedback on the status of the molt to endocrine regulators. Putative signaling peptides include MLT-8, PAN-1, and QUA-1. Features of peptide hormones present in the novel protein MLT-8 include an N-terminal secretory signal sequence, two pairs of basic amino acids suitable for proteolytic processing, and three putative N-linked glycosylation sites. The predicted MLT-8 protein also lacks motifs characteristic of association with membranes or the extracellular matrix. Thus, we expect MLT-8 to be secreted from cells where it is synthesized and to serve as a signaling molecule. Cells might also secrete PAN-1, based on predictions of an N-terminal secretory signal sequence and putative glycosylation sites.

The *qua-1 (quahog)* gene specifies a protein with a Hint domain at the C-terminus [42], a hallmark of the hedgehog family of membrane-associated intercellular signaling proteins that suggests autocatalytic cleavage [43]. The predicted QUA-1 protein possesses a secretory signal sequence, but lacks obvious sequence homology to the N-terminal domain of hedgehog, which is active in signaling [43]. The genes *ptr-4* and *ptr-23* encode proteins similar to the transmembrane transporter Dispatched [43] that are also essential for molting (Table 1) and might export QUA-1 from cells where the protein is synthesized.

The *acn-1* gene encodes a protein whose central region is 28% identical to human angiotensin converting enzyme (ACE)[32], the peptide protease that cleaves angiotensin I to angiotensin II. One model for the function of ACN-1 in molting is that ACN-1 regulates the production of a peptide molting hormone. However, ACN-1 is unlikely to directly catalyze proteolysis, because the active-site residues that coordinate zinc in human ACE are not conserved [32]. Nevertheless, ACN-1 might bind particular peptides, and thereby influence their maturation or secretion.

### Protein Synthesis

The isolation of 25 genes encoding ribosomal proteins or tRNA synthetases (Table S3) confirmed that molting requires a burst of biosynthetic activity, presumably to make components for the new cuticle [19].

### Secretion of the New Cuticle

Eighteen components of the general secretion machinery isolated in our screen (Table S2) are likely essential for synthesis of the new cuticle, including the vesicle coat proteins Sec-23p and B-cop, the small GTPase Sar-1p, and the vesicle fusion factor NSF [44, 45]. Consistent with a defect in synthesis of the new cuticle, the bodies of larvae undergoing RNAi of secretory genes often disintegrate at the molt, whereas the bodies of other Mlt larvae remain intact (unpublished data). Alternatively, defects in the secretory or endocytic trafficking of particular proteases or transmembrane proteins might account for molting defects caused by the loss of particular secretory pathway genes, similar to how the loss of the cytoplasmic adaptor protein DAB-1 interferes with molting, likely by disrupting intracellular transport of LRP-1 [46].

### Remodeling of the Exoskeleton

Many genes identified here as essential for molting encode proteins predicted to directly regulate the production or release of the collagenous cuticle. Predicted components of the cuticle include FBN-1, a protein that is 30% identical to fibrillin, the microfibril protein defective in Marfan syndrome, a common disorder of connective tissue in humans [47]. In addition, the genes *noah-1* (*nompA*-homolog) and *noah-2* encode proteins homologous to NompA, a component of specialized extracellular matrices in the fly [48]. Identification of *fbn-1, noah-1,* and *noah-2* as essential for molting suggests that incorporation of the corresponding proteins into macromolecular structures within the new cuticle might be critical for release of the cuticle at the next molt. We further identified three peroxidases likely to modify cuticle components, one of which, BLI-3, is thought to crosslink cuticle collagens [49]. Enzymatic modifications that occur after secretion of the cuticle might therefore be essential for the structural integrity of the new cuticle or shedding of the cuticle at the subsequent molt.

NAS-37 and NAS-36, two tolloid family metalloproteases independently described as essential for ecdysis [23,24], likely degrade the cuticle of the pre-molt larval stage, or regulate the maturation of other zymogens, as in the blood clotting protease cascade. NAS-37 and NAS-36 might also regulate the assembly of new cuticle [23] by processing the precursors of particular extracellular matrix proteins, just as tolloid family metalloproteases in vertebrates regulate extracellular matrix formation, in part, by cleaving the C-propeptides of procollagens [50,51]. The products of *mlt-11* and *bli-5* likely serve as extracellular protease inhibitors, because they contain multiple domains similar to bovine pancreatic trypsin inhibitor, and because a related protein from *D. melanogaster*, Papilin, localizes to the extracellular matrix in vivo and inhibits ADAMTS metalloproteases in vitro [52]. Identification of these probable anti-proteases suggests that proteolysis of the cuticle during molting is highly regulated, either spatially or temporally. In the absence of MLT-11 and BLI-5, extracellular proteases might be overly active in particular spatial domains, or at inappropriate times, a view consistent with the observation of blisters in the adult cuticle of *bli-5(RNAi)* animals (unpublished data).

The molting RNAi screen further identified transmembrane proteins expected to localize to the surface of epithelial cells and transduce signals that coordinate remodeling of the exoskeleton. The presence of MAM domains in the products of *mlt-9* and *ZC13.3* suggests a role in signaling, because the MAM domain is found in numerous transmembrane proteins of the cell-adhesion superfamily, including the receptor-like protein tyrosine phosphatase m*μ* [53]. The *lrp-1* gene, one of the first identified as essential for molting [11], and also a prominent hit in the genomic screen, encodes an LDL receptor-like protein whose intracellular domain has been proposed to function as a signaling molecule after proteolytic cleavage and release from the apical membrane of epithelial cells [54].

Taken together, the loss-of-function phenotypes and annotations of these genes suggest that the corresponding proteins regulate the formation, condensation, or degradation of the collagenous cuticle during molting. Precise transcriptional regulation of these genes as well as post-translational regulation of the corresponding gene products is likely required for the orderly synthesis and breakdown of cuticle during each molt.

In addition to the genes uncovered by RNAi, we identified the *mlt-10* gene as a hypermorphic allele in a forward genetic screen for molting mutants to be described elsewhere. The *mlt-10* gene corresponds to *C09E8.3* and represents the founding member of a large family of nematode-specific genes encoding putative membrane proteins or components of the cuticle (unpublished data).

### Remodeling of Attachments between the Muscle, Hypodermis, and Exoskeleton

*C. elegans* move by the transmission of force from the contractile apparatus of muscle to the exoskeleton via a series of lateral attachments comprised of the dense bodies and M-lines of muscle, the basement membrane situated between the muscle and the hypodermis, and hemidesmosome-like structures of the hypodermis. Consistent with the view that hemidesmosomes are remodeled at the molt, our screen identified myotactin and MUP-4 (Table S1), components of the hemidesmosomes that link, respectively, the basal membrane of the hypodermis to the basement membrane of the muscle [55], and the apical membrane of the hypodermis to the inner layer of the cuticle [56]. Surprisingly, we also isolated the muscle protein tropomyosin [57] and the basement membrane protein UNC-52 [58], suggesting that muscle attachment points might also be remodeled at the molt. Intercellular signaling involving myotactin might guide remodeling of the connections between body wall muscle and the hypodermis in much the same way myotactin maintains the association between muscle and hypodermal fibrous organelles during embryogenesis [55,59].

### Temporal and Spatial Expression Patterns of Molting Genes

We determined the spatial and temporal expression pattern of particular molting genes. We expected some of these genes to act in endocrine cells that trigger molting and some to act in epithelial cells that are remodeled during molting. Further, we anticipated much dynamic regulation during the molting cycle. Because the period between molts is short, only 8–10 h at 25 °C, we fused a PEST (Pro-Glu-Ser-Thr) signal for rapid protein degradation [60] to the C-terminus of green fluorescent protein (GFP), generating a fusion protein with a fluorescent half-life of less than 1 h in vivo. The *gfp-pest* gene was placed under the control of promoters from six genes, *nas-37, mlt-11, mlt-9, acn-1, mlt-8,* and *mlt-10*. Fusions with the conventional *gfp* gene revealed the cellular patterns, but not the detailed temporal dynamics, of expression from the promoters of *qua-1* and *xrn-2*. The genes selected for analysis encode proteins that represent the major functional categories identified in this screen; namely, regulators of gene expression, including the exoribonuclease XRN-2; putative signaling pathway components, including the hedgehog-like QUA-1, the novel secreted peptide MLT-8, and the ACE homolog ACN-1; and annotated transmembrane or cuticle proteins, including the MAM domain protein MLT-9, the protease inhibitor MLT-11, the collagenase NAS-37, and the novel protein MLT-10. Further, each gene selected for expression studies produced penetrant molting defects after RNAi, or, in the case of *mlt-10,* after mutation.

**Figure 2:**
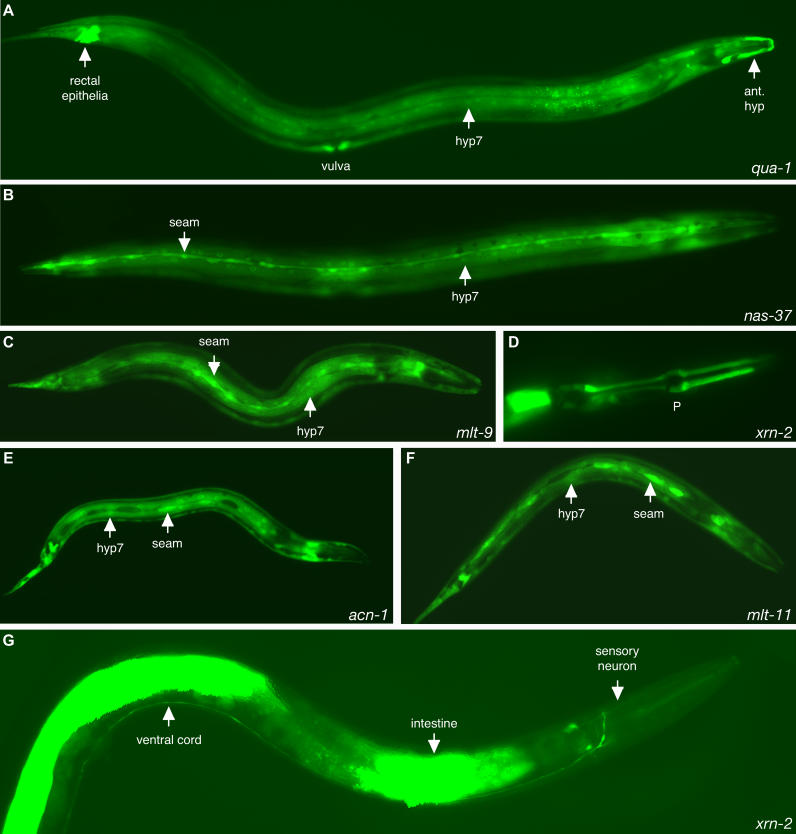
Expression of Molting Gene *gfp* Fusion Genes Expression of GFP (A,C,D,G) or GFP-PEST (B,E,F) from the promoters of the indicated genes. (A) Fluorescence from *qua-1p::gfp* in the hypodermis and specialized epithelia. (B) Fluorescence from *nas-37p::gfp-pest* in the seam cells and hypodermis of a late L4 stage larva. (C) Fluorescence from *mlt-9p::gfp* in the seam cells and hypodermis of a late L3 stage larva. (D) Fluorescence from *xrn-2p::gfp* in the pharyngeal myoepithelium (P) of a late L1 stage larva. Only the head of the worm is shown. The less intense fluorescence anterior to the posterior bulb of the pharynx likely corresponds to neurons. (E) Fluorescence from *acn-1p::gfp-pest* in the seam cells and hypodermis of a late L1 stage larva. (F) Fluorescence from *mlt-11p::gfp-pest* in the seam cells and hypodermis of a late L1 stage larva. (G) Fluorescence from *xrn-2p::gfp* in an adult worm, showing the intestine, a neuronal projection along the ventral cord, and a sensory neuron. The anterior of the worm faces right in all pictures

Fluorescence from all eight of the *gfp* fusion genes was observed in epithelial cells that synthesize cuticle (Figures 2 and S1). The *qua-1, nas-37, mlt-9*, *mlt-11, acn-1, mlt-8,* and *mlt-10* fusion genes were each expressed in the hypodermis, including the major body syncytium, hyp7, and hypodermal cells in the head and tail (Figure 2A–C and 2E–F) (unpublished data). The *nas-37, mlt-9, acn-1,* and *mlt-11* fusion genes were also expressed in the lateral seam cells (Figure 2B–C and 2E–F), which are essential for molting and morphogenesis of the cuticle [61]. Interestingly, fluorescence from *nas-37p::gfp-pest* in seam cells was observed only before the L4 stage-to-adult molt, when the cells terminally differentiate and fuse, whereas fluorescence from *mlt-9p::gfp-pest* and *mlt-11p::gfp-pest* in seam cells was observed, predominantly, before larval-to-larval molts, when the cells divide. The protease NAS-37 and anti-protease MLT-11 may therefore be required, respectively, to induce or repress fusion of the seam cells. The *gfp* fusion gene for the exoribonuclease *xrn-2* was expressed in specialized myoepithelial cells that secrete the pharyngeal cuticle (Figures 2D and S1H), consistent with the defect of *xrn-2(RNAi)* larvae in shedding cuticle from the pharynx. Particular fusion genes were also expressed in specialized interfacial cells that secrete cuticle, including the rectal epithelia (Figure 2A), the excretory duct and pore cells (Figure S1A-B), the vulval epithelium (Figure S1D), and the rectal gland (Figure S1E), as well as support cells for sensory neurons (Figure S1A and S1F).

**Figure 3:**
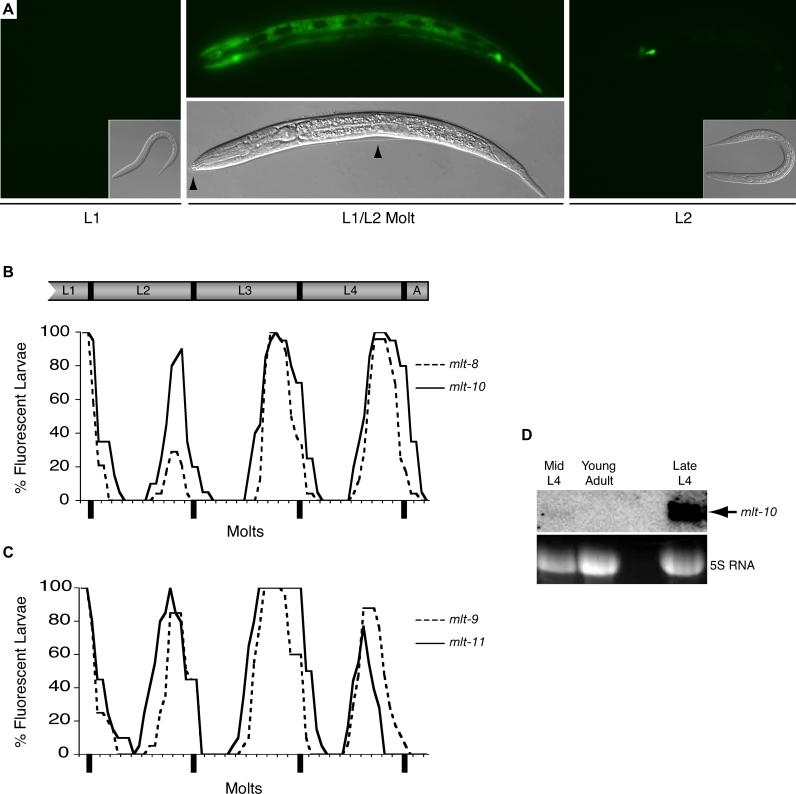
gfp Fusion Genes Are Expressed before Each Molt (A) Expression of *mlt-8p::gfp-pest* in an early L1 larva, a larva molting from the L1 to the L2 stage, and an early L2 stage larva. Black arrows indicate cuticle separated from the body of the molting larva. The particular larva shown at L2 was fluorescent before the L1/L2 molt. (B) *Ex[mlt-8p::gfp-pest]* (dashed line) or *Ex[mlt-10p::gfp-pest]* (solid line) larvae were examined for fluorescence and for molting from late in the L1 stage until early adulthood. Graph shows the percent of worms that were fluorescent over time, on a scale normalized to the molting cycle of each worm under observation (see Materials and Methods). (C) Cycles of fluorescence observed in the hypodermis and seam cells of *Ex[mlt-9p::gfp-pest]* (dashed line) or *Ex[mlt-11p::gfp-pest]* (solid line) larvae. (D) *mlt-10* messenger RNA detected by Northern analysis; ribosomal RNA stained with ethidium-bromide provides a loading control.

A pulse of fluorescence was observed in the hypodermis prior to molting, for each of the six *gfp-pest* fusion genes (Figure 3A–C) (unpublished data). Fluorescence from *mlt-8p::gfp-pest* was first detected approximately 3 h before the L1/L2 molt, or 13 h after hatchlings synchronized by starvation were fed and incubated at 25 °C. The intensity of fluorescence increased until lethargus and then decreased rapidly, such that GFP was barely detectable just 2 h after molting (Figure 3A). When monitoring individual transgenic larvae over the course of development, fluorescence from *mlt-8p::gfp-pest* was observed from 65 ± 2% to 90 ± 2% of the duration of each larval stage (see Materials and Methods). Expression of *mlt-9p::gfp-pest* and *mlt-10p*::*gfp-pest* in the hypodermis was observed at a similar time, starting, respectively, 64 ± 3% and 63 ± 2% of the way through each larval stage. In contrast, hypodermal expression of GFP from the *mlt-11* promoter was detected earlier, from 51 ± 2% to 72 ± 3% of the duration of each stage, suggesting that the MLT-11 anti-protease, synthesized midway through each larval stage, might repress proteases that are post-translationally activated at ecdysis. Expression of *mlt-9p::gfp-pest* and *mlt-11::gfp-pest* in the seam cells often preceded and lasted longer than expression in hyp7 (unpublished data). Expression of *nas-37p::gfp-pest* and *acn-1p::gfp-pest* also cycled in phase with all four molts (unpublished data). Likewise, expression of *qua-1p::gfp* in the hypodermis and of *xrn-2p::gfp* in the pharyngeal myoepithelium intensified prior to molting (unpublished data). Particular fusion genes were also expressed in the epithelial cells of late embryos that synthesize cuticle for the first larval stage. Moreover, expression of the *gfp* fusion genes was never detected in the hypodermis of gravid adults that no longer molt, whereas *mlt-10p::gfp-pest* and other fusion genes were expressed in adults that undergo a supernumerary molt due to inactivation of the heterochronic gene *lin-29* [62] (unpublished data).

To verify that cycling fluorescence from a *gfp-pest* fusion gene reflects dynamic temporal regulation of gene expression, we examined the level of *mlt-10* messenger RNA by Northern analysis. As predicted by the *mlt-10p::gfp-pest* fusion gene, the abundance of *mlt-10* mRNA in late L4 larvae exceeded that of mid L4 larvae by a factor of six, and *mlt-10* mRNA was barely detectable in young adults (Figure 3D).

Taken together, the spatial and temporal expression patterns of *mlt-8, mlt-9, mlt-10, mlt-11, nas-37, acn-1, qua-1,* and *xrn-2* indicate that the genes are expressed before molting in epithelial cells, such that the corresponding proteins are synthesized in an appropriate time and place to regulate molting. Expression of reporters for *mlt-9, mlt-10, mlt-11,* and *nas-37* in epithelial cells supported our predictions based on gene annotations that the corresponding proteins localize to either the membrane of epithelial cells or the cuticle.

Interestingly, particular fusion genes were expressed in neurons and gland cells that might produce or respond to endocrine signals regulating molting. For example, *xrn-2p::gfp* was expressed in several anterior neurons, including sensory neurons, as well as the PVT neuron that projects along the ventral cord, and the M5 pharyngeal neuron (Figures 2G, S1G, and S1H). Expression of *xrn-2* in the M5 neuron might be relevant to molting because M5 innervates gland cells whose secretions are thought to expedite release of the pharyngeal cuticle [19,35]. Also, *xrn-2p::gfp* was expressed in M5 only in larvae. The *xrn-2* reporter was also expressed in the intestine (Figure 2G), a tissue implicated in the regulation of molting as the site of synthesis of the sterol-modifying enzyme LET-767 [12]. However, expression of *xrn-2p::gfp* in the intestine persisted in adults that no longer molt, suggesting a function unrelated to molting. The *mlt-8* reporter was expressed, in larvae, in a single posterior neuron that remains to be identified. Interestingly, particular fusion genes, such as *acn-1p::gfp-pest,* were also expressed in the excretory gland cell of larvae (Figure S1A–C). The gland cell is active during ecdysis [63] and is thought to contribute material for the new surface coat. However, ablation of the excretory gland cell does not prevent molting [64], indicating that the essential function of *acn-1* in molting is unlikely to stem from expression in this cell. The neurons and other non-epithelial cells that express genes essential for molting represent candidates for foci of endocrine regulation, although the physiologic relevance of gene expression in these non-epithelial tissues remains to be determined. Analysis of the full expression patterns of these and other molting genes awaits the availability of full-length, functional *gfp* fusion genes that include intronic sequences, or antibodies against the corresponding proteins.

### Evidence of an Endocrine Cue for Molting

Observations on the expression of *mlt-10p::gfp-pest* and other reporters further support the hypothesis of an endocrine cue for *C. elegans* molting. In *nas-37(RNAi)* larvae, old cuticle often forms a natural ligature along the longitudinal axis of the worm, typically near the region of the nascent vulva. When *Ex[mlt-10p::gfp-pest] nas-37(RNAi)* larvae with such ligatures were examined late in the L4 stage, 31% (68/216) expressed GFP exclusively in hypodermis on the anterior side of the constriction, whereas no animals were fluorescent on the posterior side alone (Figure S2). 69% (148/216) of constricted larvae expressed GFP on both sides of the ligature, although in some cases fluorescence was barely detectable on the posterior side. Larvae that expressed GFP only in the anterior section stopped expressing GFP as rapidly as control larvae that completed the last molt, and, in some cases, attempted to shed the L4 cuticle from the head, indicating that the animals were not simply delayed at one point in the molting cycle (unpublished data). Further, larvae that expressed GFP only in the anterior section failed to express GFP in the posterior section up to 8 h after the normal time of the L4-to-adult molt (unpublished data), even though movement often indicated survival of the tissue on the posterior side of the ligature. Together, these observations suggest that expression of *mlt-10p::gfp-pest* and possibly molting in the posterior hypodermis requires a diffusible cue produced in the anterior of the worm. Similar experiments using man-made ligatures implicated a hormonal cue for molting of the parasitic nematode *Aphelenchus avenae* in 1967 [65]. Consistent with the view that a cue produced in the anterior of the worm stimulates molting in *C. elegans,* expression of many of the *gfp* fusion genes typically begins in the anterior hypodermis and then spreads over time to the anterior and then posterior section of the hypodermal syncytium (hyp7) (unpublished data). Also, the pre-molt cuticle first loosens from around the head during molting [19].

### Ordering Gene Expression Cascades Using *gfp* Fusion Genes

The molting cycle is a complex temporal program likely to have multiple triggers and checkpoints regulating the expression and activity of transcription factors that control multiple downstream targets. We predicted that particular genes isolated in our RNAi screen would act upstream in the molting pathway, directly or indirectly regulating the expression of genes that promote release of the exoskeleton. The availability of GFP reporters allowed us to visualize the gene regulatory status of the pathway when molting was blocked at various points by the inactivation of particular genes required for molting.

**Figure 4:**
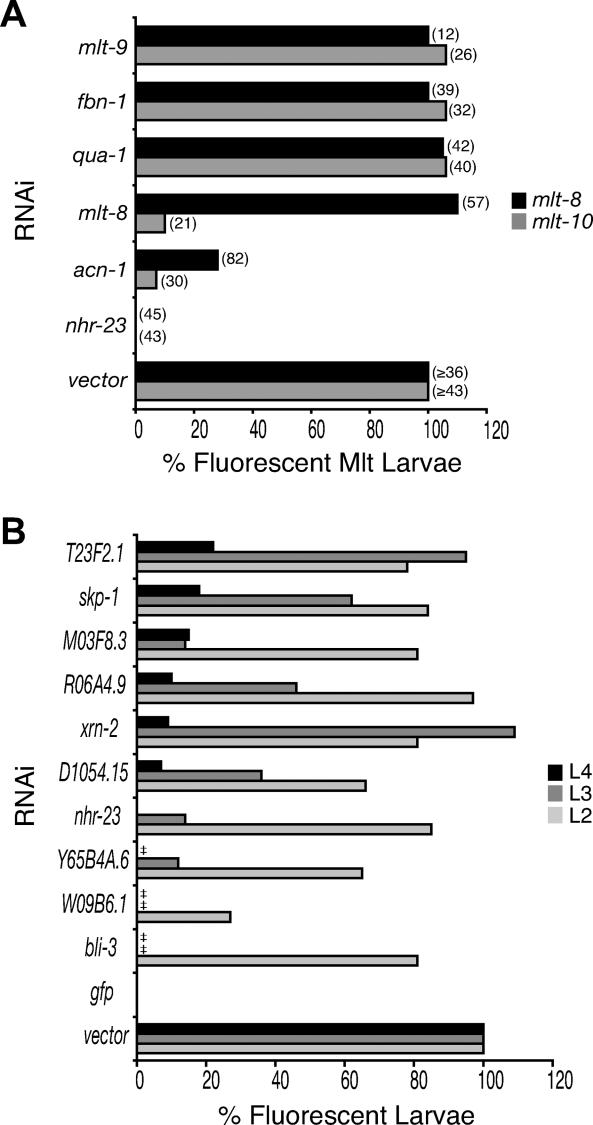
Ordering Gene Expression Cascades Using *gfp* Fusion Genes (A) *Ex[mlt-8p::gfp-pest]* or *Ex[mlt-10p::gfp-pest]* larvae were fed bacteria expressing dsRNA for each gene indicated. Graph shows the percent of animals that were fluorescent before a defective molt, normalized to the percent of control larvae that were fluorescent before molting from the same stage. The number of larvae observed is shown in parenthesis. Note that RNAi of *mlt-8* or *acn-1* typically prevented completion of the L2/L3 molt, whereas RNAi of *qua-1, fbn-1,* or *mlt-9* interfered most often with the L3/L4 or L4/A molts. RNAi of *nhr-23* blocked the L3/L4 or L4/A molts in *Ex[mlt-10p::gfp-pest]* larvae, but prevented completion of the L2/L3 molt in most *Ex[mlt-8p::gfp-pest]* larvae. In control *Ex[mlt-10p::gfp-pest]* larvae, fluorescence was observed in 95% (*n* = 56), 100% (*n* = 43), or 94% (*n* = 48) of, respectively, L2, L3, or L4 stage animals. In control *Ex[mlt-8p::gfp-pest]* larvae, fluorescence was observed in 74% (*n* = 57) or 70% (*n* = 36) of L2 stage, and 90% (*n* = 49) of L4 stage animals. Pair-wise chi-square tests indicate that the decreased fraction of *nhr-23(RNAi)* or *acn-1(RNAi)* larvae that express *mlt-8p::gfp-pest,* and of *nhr-23(RNAi), acn-1(RNAi),* or *mlt-8(RNAi)* larvae that express *mlt-10p::gfp-pest,* relative to control animals, is significant, with *p ≤* 0.001 in all cases. (B) *Ex[mlt-10p::gfp-pest]* larvae were fed bacteria expressing dsRNA for each gene indicated, or control bacteria not expressing dsRNA of a worm gene. Graph shows the percent of larvae that were fluorescent late in the late L2, L3, and L4 stage, normalized to control animals, with values representing the weighted average of two independent experiments. ‡ indicates that larvae failed to develop to the stage of observation. Table S5 contains the raw data contributing to this figure.

To order gene expression cascades among the genes uncovered by RNAi, we fed larvae expressing either the *mlt-10* or *mlt-8* reporter gene particular dsRNAs of interest. We chose to monitor fluorescence from *mlt-10p::gfp-pest* because expression of the *mlt-10* reporter likely signified the synthesis of components for the new cuticle, and because all of the transgenic animals grew vigorously and expressed GFP late in each larval stage when fed control bacteria. The *mlt-8* reporter provided a second marker expressed in the same cells at the same time. We initially examined six gene inactivations representing major functional categories identified in this screen, namely regulators of gene expression *(nhr-23),* putative signaling pathway components *(qua-1, mlt-8,* and *acn-1),* and components of the cuticle or cell membrane *(fbn-1* and *mlt-9).* Transgenic larvae fed the corresponding dsRNAs were monitored for fluorescence and molting over time (Figure 4A). Tracking individual worms ensured that expression of GFP was assessed before larval arrest ensued. Analyzing only animals that failed to molt ensured that a defect in expression of GFP would be detected even if a particular RNAi were effective in the minority of animals fed the bacterial clone.

**Figure 5:**
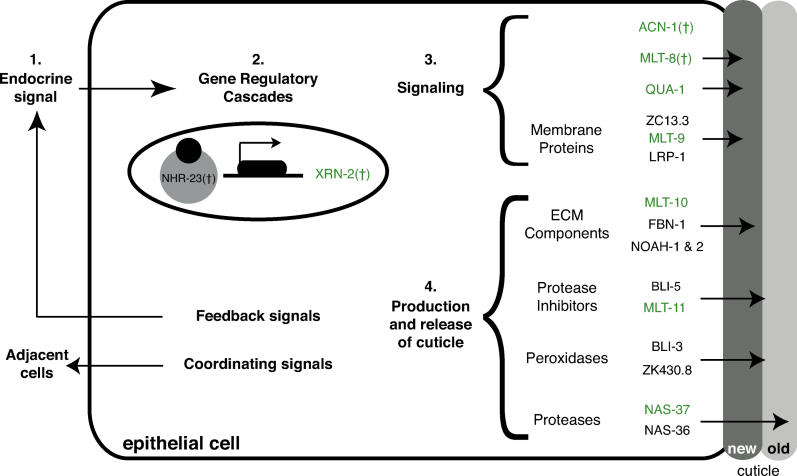
A Model for Molting of *C. elegans* (1) Endocrine and possibly neuroendocrine cues trigger molting in *C. elegans,* stimulating epithelial cells to remodel the exoskeleton near the end of each larval stage. (2) Transcriptional cascades involving NHRs alter gene expression in response to the endocrine cue. In particular, NHR-23 directly or indirectly activates expression of many genes, including *mlt-8, mlt-9, mlt-10, mlt-11, acn-1,* and *nas-37* in the hypodermis, as well as *xrn-2* in the pharyngeal myoepithelium. (3) Factors downstream of NHR-23, including MLT-8 and ACN-1, amplify the signal to molt. Signaling via transmembrane proteins likely stimulates release of the old cuticle. (4) Extracellular matrix proteins and secreted enzymes identified in our screen contribute to the new cuticle or regulate release of the old one. We expect precise regulation of these transmembrane proteins and secreted enzymes to accompany the molt. In theory, intercellular signaling might coordinate events in different epithelial cells, the muscle, and the intestine. We further expect secreted signals to provide feedback on the status of the molt to endocrine regulators. The Hint domain protein QUA-1 is a good candidate for a signal secreted from the hypodermis that might amplify a cue for ecdysis, signal to adjacent tissues, or provide feedback. Green shading indicates that a *gfp* fusion to the corresponding gene was expressed in epithelial cells. † indicates that the gene is required for expression of *mlt-10p::gfp-pest* in the hypodermis*.*

Figure 4A shows that all *nhr-23(RNAi)* animals failed to express GFP from either the *mlt-10* or *mlt-8* promoter prior to their ill-fated molt. Inactivation of *nhr-23* also diminished expression of the reporters for *nas-37, mlt-11, mlt-9, acn-1,* and *qua-1* in the hypodermis, and of the *xrn-2* reporter in the pharyngeal myoepithelium (unpublished data). Thus, NHR-23, synthesized in epithelial cells [13], likely initiates or sustains the pulse of *mlt* gene expression late in each larval stage, perhaps provoking a response to an as-yet unidentified molting hormone (Figure 5). Inactivation of the *acn-1* and *mlt-8* genes likewise abrogated expression of GFP from the *mlt-10* promoter (Figure 4A), suggesting that ACN-1 and MLT-8 function upstream of *mlt-10* but downstream of NHR-23 in this regulatory cascade.

In contrast, inactivation of the hedgehog-like gene *qua-1,* the *fibrillin* homolog *fbn-1,* or the MAM domain gene *mlt-9* produced larvae that expressed GFP from both the *mlt-10* and *mlt-8* promoters, but nevertheless failed to complete ecdysis (Figure 4A); indicating that these three genes are dispensable for expression of the *mlt-10* and *mlt-8* reporters, and likely to function downstream of, or in parallel to, the *mlt-10* gene (Figure 5).

To identify additional points of transcriptional control, populations of *Ex[mlt-10p::gfp-pest]* larvae were fed bacteria expressing dsRNAs corresponding to 76 genes uncovered in our screen, and then monitored for fluorescence late in the L2, L3, and L4 stages. Inactivation of the genes *xrn-2, Y65B4A.6, skp-1, D1054.15, R06A4.9, W09B6.1, M03F8.3, T23F2.1,* and *crs-2,* in addition to *nhr-23, acn-1, and mlt-8,* significantly (*p* ≤ 0.001) abrogated expression of GFP during a particular stage and blocked development shortly thereafter (Figures 4B and S3; Table S5), suggesting that the corresponding proteins normally induce or sustain expression of *mlt-10.* The genes identified as putative regulators of the *mlt-10* gene encode, respectively, the 5′-3′ exoribonuclease XRN-2, a DEAD (Asp-Glu-Ala-Asp) box helicase, a putative co-factor of NHR-23 [30], two WD-beta repeat proteins, the enzyme acetyl-Coenzyme A carboxylase, a homolog of the spliceosome-associated factor CRN1 [66,67], a glycosyltransferase, and a cysteinyl tRNA synthetase. The *xrn-2* gene was verified as a positive regulator of *mlt* gene expression by tracking fluorescence from individual transgenic larvae over time (unpublished data). Together, we expect the 12 genes upstream of *mlt-10p::gfp-pest* to be required for epithelial cells to initiate or maintain remodeling of the exoskeleton during molting.

Inactivation of other genes did not significantly (*p* > 0.001) reduce expression of GFP relative to control larvae of the same stage (Table S5). In most cases, the formal possibility that the corresponding proteins regulate the *mlt-10* gene cannot be eliminated due to the variable efficacy of RNAi, particularly if inactivation of a gene partly reduced expression of GFP. However, inactivation of the genes *bli-3* (Figure 4B)*, nas-37, lrp-1, bli-5, ZK430.8, unc-52, lev-11, W10G6.3, kin-2, bli-1, gei-16, K04A8.6,* and *F25H8.6* produced molting-defective larvae that expressed GFP (unpublished data), demonstrating with certainty that these gene activities are not necessary for expression of *mlt-10p::gfp-pest* and suggesting, instead, that the genes act downstream of, or in parallel to, the *mlt-10* gene. Gene products dispensable for *mlt-10* expression might act very near, or, at the time of ecdysis, consistent with our predictions based on gene annotations that the products of *fbn-1, bli-3,* and *ZK430.8* promote assembly or modification of the cuticle. Monitoring expression of the *gfp* fusion genes thus allowed a first sorting of molting genes uncovered by RNAi into pathways.

## Discussion

Using functional genomics, we identified a large set of genes essential for molting in *C. elegans*. Figure 5 shows a model for the regulation of molting where endocrine or neuroendocrine cues generated by as-yet unidentified cells trigger epithelial cells to remodel the exoskeleton at the end of each larval stage. Particular genes uncovered in this screen encode proteins that regulate gene expression during the molting cycle, whereas other genes encode signaling molecules likely to coordinate the multicellular process of molting. Together, gene annotations as well as spatial and temporal expression studies suggest that many genes identified here specify transmembrane proteins, secreted enzymes, and structural components of the cuticle that are synthesized in epithelial cells and likely regulate the de novo production or release of the exoskeleton at each molt. Thus, activation of the anti-protease MLT-11, the collagenase NAS-37, the MAM domain protein MLT-9, or the LDL-receptor-like protein LRP-1 each represents a potential focus for the spatial and temporal regulation of ecdysis.

Fusions to GFP show that expression of several genes uncovered in this screen cycles in phase with molting, similar to the expression of genes encoding particular NHRs and cuticle collagens [68,69]. Analysis of GFP reporters shows further that NHR-23, directly or indirectly, activates expression of many genes, including *mlt-8, mlt-9, mlt-10, mlt-11, nas-37,* and *acn-1* in the hypodermis, as well as *xrn-2* in the pharyngeal myoepithelium. NHR-23 is also required for expression of a cuticle collagen gene, *dpy-7* [14], whose product is incorporated into each larval cuticle [69]. Thus, the receptor coordinates gene expression in epithelial cells, possibly in response to an endocrine cue for molting. A ligand for NHR-23 has yet to be identified, but the molecule could be synthesized in neuroendocrine cells or in steroidogenic cells coupled to neurons that regulate molting. Production of a ligand for NHR-23 is likely to be tightly regulated, similar to how steroidogenesis in the prothoracic gland of insects is induced by the neuropeptide prothoracicotropic hormone but also repressed by ecdysteroids [3,70].

Our screen identified the exoribonuclease XRN-2 as a novel regulator of gene expression during molting. One model is that XRN-2 down-regulates the abundance of protein-coding mRNAs or microRNAs that correspond to negative regulators of molting. Together, the observations that *xrn-2(RNAi)* larvae fail to shed the pharyngeal cuticle and that *xrn-2p::gfp* is expressed in the pharyngeal myoepithelium suggest that XRN-2 promotes molting in the pharynx. However, *xrn-2* is also required for expression of the *mlt-10* reporter in the hypodermis, a tissue where expression of *xrn-2p::gfp* itself has not been detected. One possibility is that XRN-2 activity in the pharynx leads to an intercellular cue that promotes expression of genes in hyp7. Alternatively, *xrn-2* might be expressed in the hypodermis, but might not be detectable using this particular *gfp* fusion gene. XRN-2 and the product of *Y65B4A.6,* another gene isolated in this screen, might work together to regulate gene expression, because *Y65B4A.6* encodes a DEAD-box helicase, and a DEAD-box helicase functions along with the Xrn1p/Rat1p exoribonuclease in mRNA degradation in yeast [71]. Together, the requirement for *xrn-2* in molting and for the related gene *xrn-1* in embryogenesis [72], establish the XRN family of exoribonucleases as important developmental regulators in *C. elegans*.

How the multiple genetic pathways uncovered by RNAi converge to regulate gene expression during the molting cycle of *C. elegans* remains to be determined. However, one or more of these pathways might couple progression of the molting cycle to physiologic or environmental cues including the nutritional status of the larva.

Our identification of putative signaling molecules suggests an essential role for intercellular communication in molting of *C. elegans*. One idea is that signaling between different epithelia, such as the hypodermal syncytium and the lateral seam cells, might coordinate the production or release of cuticle. Consistent with this view, transcription factors regulating the differentiation and fusion of seam cells are also required for molting [61]. Signaling between the hypodermis and muscle might coordinate remodeling of hemidesmosomes and muscle attachment points. In theory, intercellular signaling might also coordinate division or fusion of the seam cells or endoreduplication of intestinal nuclei with the molt. An alternative view is that cell-autonomous responses to one or a few endocrine cues account for the coordinated activities of different cell types during molting of *C. elegans,* similar to how different tissues respond to changes in the titer of 20-hydroxyecdysone during insect metamorphosis [2,73].

Particular secreted peptides identified in our screen might amplify endocrine cues for molting. The novel peptide MLT-8 might serve as an autocrine cue that sustains production of the new cuticle, because *mlt-8* promotes expression of the GFP reporter for *mlt-10* in the same epithelial cells where MLT-8 itself is synthesized. Also, the Hint domain protein QUA-1 is a good candidate for a signal secreted from the hypodermis that could generate a spatially patterned response in the hypodermis itself or in adjacent tissues, thereby coordinating the final stages of the molt.

To set the molting cycle, we expect secreted signals from epithelial cells to provide feedback on the status of the molt to endocrine or neuroendocrine regulators. The existence of physiologic feedback cues is consistent with the observation that many larvae that fail to ecdyse also arrest development, including those larvae defective in proteins that function in epithelial cells, such as LRP-1 [11]. Interference with ecdysone signaling in epidermal tissues similarly triggers a global arrest during *Drosophila* metamorphosis, suggesting the existence of a molting “checkpoint” in insect development [74]. In theory, any of the signaling components isolated in this screen might function in feedback pathways active during one or more steps of the molt.

Particular peptide hormones might also function in neuroendocrine circuits that regulate the quiescence of larvae during lethargus or the behaviors characteristic of ecdysis, in much the same way that peptide hormones of insects trigger behaviors essential for escape from the old exoskeleton [4,8]. However, none of the putative secreted peptides identified in this screen show obvious sequence similarity to eclosion hormone or ecdysis-triggering hormone.

Genes or hormones that function far upstream in the molting pathway of nematodes can now be identified, respectively, as mutations or compounds that alter the timing of expression of the cycling GFP reporters. Master regulators of molting in nematodes might function in endocrine or possibly neuroendocrine cells and might be conserved in arthropods, given that molting is a universal feature of the Ecdysozoan clade [1]. One simple explanation for the abundance of epithelial, as opposed to neuronal, genes uncovered in the screen described here is that RNAi works better in epithelial cells than in neurons [75]. Particular gene inactivations that produced molting defects at low penetrance in our screen (Table S4) might therefore correspond to endocrine or neuroendocrine components. In addition, a screen for arrest during ecdysis, rather than a screen for aberrant timing of the molt, might enrich for epithelial factors.

Identifying genes essential for molting of *C. elegans* enables the development of safe and effective insecticides and nematicides that target gene products conserved only in Ecdysozoans. Molting genes conserved only in insects and nematodes, such as the extracellular matrix proteins NOAH-1 and NOAH-2, identify potential targets for insecticides expected to harm only Ecdysozoans. Current anti-nematode drugs, such as benzimidazoles and avermectins, target, respectively, cytoskeletal components and ion channels that are conserved in mammals, and the drugs therefore can be toxic to humans. Resistance to these compounds is also increasingly common [76,77]. One potential new drug target is MLT-8, since the corresponding gene is conserved and highly expressed at the molt in a parasitic nematode, as inferred by the identification of 32 cDNAs matching *C. elegans mlt-8* (*p* = E^-^121) in a library derived from molting *O. volvulus* (Table S7) (unpublished data). However, the novelty of MLT-8 may pose a considerable challenge for drug development. In this regard, molting proteases, like NAS-37, represent more attractive targets for the development of small-molecule antagonists, given the success of drug development on protease targets for high blood pressure and HIV [78,79].

## Materials and Methods

### Screening the RNAi library for molting genes

Approximately 16,757 bacterial clones that each express a 0.5- to 2-kilobase dsRNA corresponding to one worm gene were obtained courtesy of J. Ahringer's laboratory [26,27]. An additional 1,821 bacterial clones that express dsRNAs corresponding to worm genes not represented in the Ahringer library were obtained courtesy of M. Vidal [28]. Bacterial clones expressing dsRNA of worm genes were cultured as described [26], except that nematode growth medium was supplemented with 8 mM IPTG and 25 μg/ml carbenicillin. Approximately 25 wild-type (N2) hatchlings (early L1 larvae) were isolated using standard techniques, fed a particular bacterial clone, and cultivated for 2.5 d at 20 °C before visual inspection. Although animals were examined blind to the identity of the dsRNA, the library designation for each bacterial clone could be readily decoded to reveal the corresponding gene [27]. Gene inactivations observed to cause molting defects in the primary library screen were tested again by feeding the bacterial clones to about 50 wild-type (N2) or 50 *rrf-3(pk1426)* mutant larvae [29]. In a control, larvae with molting defects were not observed after about 1,000 N2 or *rrf-3(pk1426)* animals were fed isogenic bacteria not expressing dsRNA of worm genes. Also, the vast majority of *E. coli* strains expressing dsRNAs of particular worm genes caused no molting defects. To verify the identity of genes inactivated by RNAi, plasmid DNA was isolated from each bacterial clone of interest and the insert DNA sequenced using primers complementary to the vector pPD129.36 [80]. In this screen, 7% of clones contained worm DNA different from the expected insert. Sequence and protein names refer to designations by WormBase (http://www.wormbase.org/).

To evaluate the dauer molt, approximately 20 hatchlings of the temperature-sensitive, dauer-constitutive mutants *daf-2(e1370)* [81] and *daf-7(e1372)* [82] were fed bacterial clones expressing dsRNA for each molting gene, in duplicate, and then cultivated at restrictive temperature (25 °C) for 3 d, such that control animals all became dauers. Animals were then shifted to permissive temperature (15 °C) for 2 d, allowing control animals to molt to the L4 stage. Observation of L2d or dauer larvae with the Mlt phenotype, in either genetic background, indicated that a given gene inactivation disrupted the L2d/dauer or dauer/L4 molt (Table S6).

### Strains and molecular constructs

Strains used for this study appear in Table S8. Table S9 shows the PCR primers used to construct *gfp* fusion genes. To append the PEST sequence to the C-terminus of GFP, nucleotides 1,399–1,521 of pd1EGFP-N1 (Clontech, Palo Alto, California, United States) were inserted into pPD95_81 (A. Fire) between the last coding codon and the stop codon of *gfp,* generating pAF207. For each gene, primers U1 and FL were used to amplify N2 genomic DNA corresponding to the initiation codon and upstream sequence, while primers FU and CAW31 (5′- GCCGCATAGTTAAGCCAGCC 3′) [83] were used to amplify DNA corresponding to the *gfp-pest* or *gfp* gene and the 3′ UTR of *unc-54* from, respectively, pAF207 or pPD95_81. The PCR products were annealed and the resulting polynucleotide amplified using primers U2 and CAW32 (5′ CCGCTTACAGACAAG CTGTGA 3′) under conditions described previously [83]. To generate the extrachromosomal arrays *mgEx646, mgEx647, mgEx648, mgEx649, mgEx656, mgEx654, mgEx650, mgEx675,* and *mgEx676,* PCR products corresponding to, respectively, *mlt-10p::gfp-pest, mlt-8p::gfp-pest, mlt-9p::gfp-pest, mlt-11p::gfp-pest, nas-37p::gfp-pest, acn-1p::gfp-pest, mlt-9p::gfp, qua-1p::gfp,* and *xrn-2p::gfp* were microinjected at 5 to 10 ng/μl into temperature-sensitive *pha-1(e2123)* mutant animals along with the *pha-1(+)* plasmid pBX [84] at 3 ng/μl and pBS DNA to a final concentration of 100 ng/μl. Use of *the pha-1(e2123)* genetic background allowed for the recovery and cultivation of worm populations in which virtually all of the animals maintained the extrachromosomal array, because only *pha-1(+)* transgenic embryos survive at 25 °C [84]. Verifying that GFP-PEST fusion proteins are degraded by the proteosome, we found that RNAi of proteosome subunit genes like *pbs-5* prolonged fluorescence from *mlt-10p::gfp-pest* even in developmentally arrested larvae (Table S5 and Figure S3). Fusions between the conventional *gfp* gene and the promoters of *qua-1* and *xrn-2* were used for expression studies because fluorescence was not readily detected from fusions to the *gfp-pest* gene.

### Comparative sequence searches

Sequence homologs of the MLT proteins were identified using the standard algorithm TBlastN [85] to compare the predicted product of each molting gene to the GenBank library of translated DNA sequences from *H. sapiens, D. melanogaster,* and *S. cerevisiae,* or translated cDNA sequences from nematodes. The most highly related proteins from human, fly, or yeast were then compared against all worm proteins using the algorithm BlastX and the WormPep database at WormBase (http://www.wormbase.org). Signal peptides were identified in predicted gene products using either the SignalP 3.0 server (http://www.cbs.dtu.dk/services/SignalP/) or Kyte-Doolittle Hydropathy plots.

### Monitoring *gfp* fusion gene expression

To monitor temporal expression of the *mlt* gene *gfp-pest* fusion genes, synchronized L1 hatchlings of GR1348, GR1349, GR1350, or GR1351 (Table S8) were plated on nematode growth medium with *E. coli* strain OP50 as a food source and incubated at 25 °C. Fluorescent larvae were selected 14 h later to ensure the use of non-mosaic, highly synchronous animals. Each larva was scored every hour for detectable fluorescence, using a Zeiss (Oberkochen, Germany) Stemi-SV6 microscope, and for molting, indicated by shedding of the cuticle. Each animal was transferred to a new plate after each molt. In Figure 3, we report the percent of animals that were fluorescent over time, on a scale normalized to the period between molts for each worm under observation. As an example, a larva that molted from L1 to L2 at noon, molted from L2 to L3 at 8 P.M., was fluorescent at 7 P.M. and 8 P.M., but was not fluorescent at 6 P.M. or 9 P.M., would be recorded as fluorescent from time 1.87 to time 2.0, or, from 87.5% to 100% of the L2 stage. Calculations of the average duration of fluorescence, reported with the 95% confidence interval, include observations from larvae during the L2, L3, and L4 stages. Because many of the extrachromosomal arrays were associated with some larval lethality, only larvae that completed all four molts were included in the final analysis. Twenty-four larvae were observed for *mlt-8p::gfp-pest* whereas 20 larvae were observed for all other reporters. For northern analysis, RNA from extracts of mid L4, late L4, and young adult animals was resolved and hybridized with a *mlt-10* probe, corresponding to base pairs 5,070 to 6,997 of cosmid C09E8, as previously described [86]. Message levels were quantified using ImageQuant software and a phosphorimager.

To order gene expression cascades, synchronized hatchlings of *mgEx646[mlt-10p::gfp-pest]* (GR1348) and *mgEx647[mlt-8p::gfp-pest]* (GR1349) were fed bacteria expressing dsRNA for each gene of interest, or, as a control, fed isogenic bacteria not expressing dsRNA for a worm gene. After incubation for no more than 15 h at 25 °C, a fluorescent larva was transferred to each well of several 24-well RNAi plates seeded with the appropriate bacteria. For each developmental stage, larvae were observed over a 6 to 9 h time period starting when control larvae first became fluorescent, and scored every 2–3 h for detectable fluorescence and for the Mlt phenotype. In Figure 4A, we report the percent of animals that were fluorescent prior to a defective molt, normalized to the fraction of control larvae that were fluorescent before molting from the same stage. To screen the full set of molting gene inactivations, approximately 20 synchronized hatchlings of GR1348 were fed bacteria expressing dsRNA corresponding to each gene of interest in two trials. The percent of larvae with detectable fluorescence was scored 1–3 h before control larvae molted from the L2, L3, or L4 stage, when the majority of control larvae were fluorescent. Only larvae of the same developmental stage as control animals were scored at each time point. To examine patterns of GFP expression in *nas-37(RNAi) mgEx646[mlt10p::gfp-pest]* larvae, hatchlings of GR1348 were fed bacteria expressing dsRNA for *nas-37* and L4 stage larvae examined after 39–43 h of growth at 25 °C. Larvae with coronal constrictions were scored for fluorescence using a Zeiss M2-Bio microscope. Twenty-six larvae that expressed GFP only anterior of the ligature, and 22 fluorescent control larvae, were then transferred to new plates and each worm was observed for fluorescence and molting 4 and 8 h later.

## Supporting Information

Figure S1 Expression of Molting Gene *gfp* Fusion Genes (A–C) Expression of *acn-1p::gfp-pest.* (A) Fluorescence in the excretory gland, duct, and pore cells (Exc), and in the glial cells (Gi) of interlabial neurons. (B) Nomarski image of the same larva. (C) Expression in the excretory gland cell (Gn). (D and E) Expression of *nas-37p::gfp-pest* in specialized epithelia. (D) Expression in the vulva. (E) Fluorescence in the rectal gland, labeled RG. The solid line traces the tail of the worm, and the dashed line shows the edges of the intestine. (F) Expression of *qua-1p::gfp* in support cells for head neurons. (G and H) Expression of *xrn-2p::gfp.* Expression in the M5 pharyngeal neuron (G) and posterior bulb of the pharynx (H) of the same larva. (C), (D), and (F) show fluorescence images superimposed on Nomarski optics. P, posterior bulb of the pharynx.
(26 MB EPS).

Figure S2 Effect of Ligatures on Expression of *mlt-10p::gfp-pest*
Patterns of GFP expression in *nas-37(RNAi) Ex[mlt10p::gfp-pest]* larvae observed at the L4 stage. Diagrams show the anterior (A) of the worm facing left, and green color indicates expression of GFP. 216 larvae selected for observation at the L4 stage were constricted by old cuticle from a prior larval stage. The number of larvae observed in each class is shown in parenthesis. Approximately 7% (*n* = 1,109) of animals lacking a ligature showed more intense expression of GFP in hypodermal tissues anterior of the nascent vulva at the time of observation.
(3.2 MB EPS).

Figure S3 Expression of *mlt-10p::gfp-pest* After RNAi of Genes Important for Molting *Ex[mlt-10p::gfp-pest]* larvae were fed bacteria expressing dsRNA for each gene indicated, or control bacteria not expressing dsRNA of a worm gene. Graph shows the percent of larvae that were fluorescent in the late L4 stage, normalized to control animals. Values represent the weighted average of two independent trials, with an average of 38 larvae examined per bacterial clone. Asterisks indicate trials where the fraction of fluorescent larvae differed significantly from that of controls (*p* ≤ 0.001 in pair-wise chi-square tests). Table S5 contains the raw data contributing to this figure.
(735 KB EPS).

Table S1 Genes Whose Inactivation Disrupts Molting and Their Relation to Genes in Other Species (1) Sequence names as designated by WormBase (http://www.wormbase.org/)
(2) Top hits from TBlastN searches of the human or fly genome using the predicted *C. elegan*s gene product. Red shading of the text indicates that a BlastX search with the predicted human or fly protein uncovered the corresponding *C. elegans* protein as the top-scoring match in *C. elegans*, suggesting homology.
(56 KB XLS).

Table S2 Genes Required for General Secretion and Endocytosis Uncovered in the Molting Screen (19 KB XLS).

Table S3 Genes Required for Protein Synthesis Uncovered in the Molting Screen (20 KB XLS).

Table S4 Gene Inactivations Producing Molting Defects in 10% or Less of Larvae (23 KB XLS).

Table S5 Expression of *mlt-10p::gfp-pest* during RNAi of Molting Genes * Vector B is the control sample for RNAi of *K04A8.6, ZC13.3, ZK945.2, C15H11.7, F32D8.6, T19A5.3, Y65B4A.6, Y23H5A.1, R06A4.9, Y47D3B.1, Y54E10BR.5, T25B9.9, T17H7.4, Y105E8B.1, W03F9.10, F56C9.1, C23G10.10,* and *F25H8.6*. Vector A is the control for all other gene inactivations.
N.A., not applicable (RNAi caused larval arrest at an earlier stage); N.D., not determined; #, RNAi produced larvae arrested at the L2 to L3 stage that continued to express GFP at this time point.
(53 KB XLS).

Table S6 Inactivation of *mlt* Genes in Dauer Larvae + indicates the observation of dauer larvae trapped in cuticle among approximately 40 of the dauer-constitutive conditional mutants *daf-2(e1370)* or *daf-7(e1372)*. Hatchlings were incubated at 25 °C to drive dauer formation, and then at 15 °C to allow recovery and a molt to the L4 stage. Note that dsRNAs that cause a larval arrest at the L1 or L2 stage (Table S5) could not be evaluated using this method.
(22 KB XLS).

Table S7 Homologs of Selected Molting Genes in Parasitic Nematodes ^1^ Top hits and scores from TBlastN searches with the predicted *C. elegans* gene product versus translated cDNAs isolated from the indicated species.
(79 KB XLS).

Table S8 Strains Used in This Study (17 KB XLS).

Table S9 Primers Used for Construction of *gfp* Fusion Genes R1 refers to the sequence 5′- CGGGATTGGCCAAAGGACCCAAAG-3′; R2 refers to the sequence 5′- CTTTGGGTCCTTTGGCCAATCCCG-3′
(19 KB XLS).

### Accession Numbers

The GenBank (http://www.ncbi.nlm.nih.gov/Genbank) accession number for cosmid C09E8 is AF077529.

The WormBase (http://www.wormbase.org/) accession numbers for genes and gene products discussed in this paper are *acn-1* (C42D8.5), *bli-1* (C09G5.6), BLI-3 (WP:CE28463), *bli-4* (K04F10.4), *bli-5* (F45G2.5), *crs-2* (Y23H5A.1), DAB-1 (WP:CE36446), *dpy-7* (F46C8.6), *fbn-1* (ZK783.1), *gei-16* (T17H7.4), *kin-2* (R07E4.6), LET-767 (WP:CE30639), *lev-11* (Y105E8B.1), LIN-26 (WP:CE27972), *lin-29* (W03C9.4), *lir-1* (F18A1.3), *lrp-1* (F29D11.1), LRP-1 (WP:CE05765), *mlt-8* (W08F4.6), *mlt-9* (F09B12.1), *mlt-10* (C09E8.3), *mlt-11*(W01F3.3), *nas-36* (C26C6.3), *nas-37* (C17G1.6), *nhr-23* (C01H6.5), NHR-23 (WP:CE24775), *nhr-25* (F11C1.6), NHR-25 (WP:CE03191), *noah-1* (C34G6.6), *noah-2* (F52B11.3), PAN-1 (WP:CE01041), PEB-1 (WP:CE07500), *ptr-23* (ZK270.1), *ptr-4* (C45B2.7), *qua-1* (T05C12.10), *rme-8* (F18C12.2), *skp-1* (T27F2.1), *unc-52* (ZC101.2), *xrn-1* (Y39G8C.1), and *xrn-2* (Y48B6A.3).

## Abbreviations

ACE: angiotensin converting enzyme
GFP: green fluorescent protein
LDL: low-density lipoprotein
NHR: nuclear hormone receptor
RNAi: RNA-interference
